# Characterization of *Diaporthe* species on *Camelliaoleifera* in Hunan Province, with descriptions of two new species

**DOI:** 10.3897/mycokeys.84.71701

**Published:** 2021-10-18

**Authors:** Qin Yang, Jie Tang, Guo Y. Zhou

**Affiliations:** 1 Forestry Biotechnology Hunan Key Laboratories, Central South University of Forestry and Technology, Changsha 410004, China Central South University of Forestry and Technology Changsha China; 2 The Key Laboratory for Non-Wood Forest Cultivation and Conservation of the Ministry of Education, Central South University of Forestry and Technology, Changsha 410004, China Central South University of Forestry and Technology Cahngsha China

**Keywords:** *Camelliaoleifera*, DNA phylogeny, systematics, taxonomy, two new taxa

## Abstract

Tea-oil tree (*Camelliaoleifera* Abel.) is an important edible oil woody plant with a planting area over 3,800,000 hectares in southern China. Species of *Diaporthe* inhabit a wide range of plant hosts as plant pathogens, endophytes and saprobes. At present, relatively little is known about the taxonomy and genetic diversity of *Diaporthe* on *C.oleifera*. Here, we conducted an extensive field survey in Hunan Province in China to identify and characterise *Diaporthe* species associated with tea-oil leaf spots. As a result, eleven isolates of *Diaporthe* were obtained from symptomatic *C.oleifera* leaves. These isolates were studied by applying a polyphasic approach including morphological and phylogenetic analyses of partial ITS, *cal*, *his3*, *tef1* and *tub2* gene regions. Two new *Diaporthe* species (*D.camelliae-oleiferae* and *D.hunanensis*) were proposed and described herein, and *C.oleifera* was revealed to be new host records of *D.hubeiensis* and *D.sojae*. This study indicated there is a potential of more undiscovered *Diaporthe* species from *C.oleifera* in China.

## Introduction

Tea-oil tree, *Camelliaoleifera* Abel., is a unique woody edible oil species in China, mainly distributed in the Qinling-Huaihe River area. It has a long history of cultivation and utilization for more than 2300 years since ancient China (Zhuang 2008). Camellia oil, obtained from *C.oleifera* seeds, is rich in unsaturated fatty acids and unique flavors, and has become a rising high-quality edible vegetable oil in China. The edible of tea-oil is also conducive to preventing cardiovascular sclerosis, anti-tumor, lowering blood lipid, protecting liver and enhancing human immunity (Wang et al. 2007). Hunan Province leads the country in *C.oleifera* production with the average of 3.3~40,000 hm^2^ to expand the cultivation area every year (Tan et al. 2018). By the end of 2017, the cultivation area of *C.oleifera* reached 1.4 million hm^2^, tea oil 290100 tons, and output value of 35 billion yuan (Tan et al. 2018). Thus, the development of *C.oleifera* industry is of great significance for the economic development of Hunan Province and the poverty alleviation of local farmers.

Diseases are a major constraint to *C.oleifera* production. Anthracnose disease caused by *Colletotrichum* species is one of the foremost diseases in southern China, which can infect leaves and fruits of *C.oleifera*, causing up to 40% fruit drop and up to 40% camellia seeds loss (Wang et al. 2020). During July and August of 2020, new leaf spots were detected on tea-oil tree with irregular, brownish-grey lesions, often associated with leaf margins. Infected leaves cultured on medium had dark pycnidia producing ellipsoid guttulate conidia, similar to that of *Diaporthe* species (Yang et al. 2020, 2021). *Diaporthe* species are responsible for diseases on a wide range of plant hosts, including agricultural crops, forest trees and ornamentals, some of which can cause substantial yield losses (Santos et al. 2011; Gomes et al. 2013; Udayanga et al. 2015; Gao et al. 2016; Guarnaccia and Crous 2017, 2018; Yang et al. 2018, 2020, 2021). For instance, *D.ampelina*, the causal agent of Phomopsis cane and leaf spot, is known as a severe pathogen of grapevines (Hewitt and Pearson 1988), infecting all green tissues and causing yield reductions of up to 30% in temperate regions (Erincik et al. 2001). *Diaporthecitri* is another well-known pathogen exclusively found on *Citrus* spp. causing melanose, stem-end rot and gummosis in all the citrus production area except Europe (Mondal et al. 2007; Udayanga et al. 2014a; Guarnaccia and Crous 2017, 2018).

Species identification criteria in *Diaporthe* has mainly relied on host association, morphology and culture characteristics (Mostert et al. 2001; Santos and Phillips 2009; Udayanga et al. 2011), which resulted in the description of over 200 species. Some species of *Diaporthe* were reported to colonise a single host plant, while other species were found to be associated with different host plants (Santos and Phillips 2009; Diogo et al. 2010; Santos et al. 2011; Gomes et al. 2013). In addition, considerable variability of the phenotypic characters was found to be present within a species (Rehner and Uecker 1994; Mostert et al. 2001; Udayanga et al. 2011). During the past decade, a polyphasic approach, based on multi-locus DNA data, morphological, phytopathological and phylogenetical analyses, has been employed for species boundaries in the genus *Diaporthe* (Huang et al. 2015; Gao et al. 2016, 2017; Guarnaccia and Crous 2017; Guarnaccia et al. 2018; Yang et al. 2018, 2020, 2021).

The classification of *Diaporthe* has been ongoing; however, little is known about species able to infect *C.oleifera*. Thus, the objective of the present study was to identify the prevalence of *Diaporthe* spp. associated with tea-oil tree leaf spot in the major plantations in Hunan Province based on morphological and phylogenetic features.

## Materials and methods

### Fungal isolation

Leaves of *C.oleifera* with typical symptoms of leaf spots were collected from the main tea-oil camellia production fields in Hunan Province. Small sections (3 × 3 mm) were cut from the margins of infected tissues, and surface-sterilised in 75% ethanol for 30 s, then sterilised in 5% sodium hypochlorite for 1 min, followed by three rinses with sterilised water and finally dried on sterilised filter paper. The sections were then plated on to PDA plates and incubated at 25 °C. Fungal growth was examined daily for up to 7 d. Isolates were then transferred aseptically to fresh PDA and purified by single-spore culturing. All fungal isolates were placed on PDA slants and stored at 4 °C. Specimens and axenic cultures are maintained in the Central South University of Forestry and Technology (CSUFT).

### Morphological and cultural characterization

Agar plugs (6 mm diam.) were taken from the edge of actively growing cultures on PDA and transferred on to the centre of 9 cm diam. Petri dishes containing 2% tap water agar supplemented with sterile pine needles (PNA; Smith et al. 1996) and potato dextrose agar (PDA), and incubated at 25 °C under a 12 h near-ultraviolet light/12 h dark cycle to induce sporulation as described in recent studies (Gomes et al. 2013; Lombard et al. 2014). Colony characters and pigment production on PNA and PDA were noted after 10 d. Colony colours were rated according to Rayner (1970). Cultures were examined periodically for the development of ascomata and conidiomata. The morphological characteristics were examined by mounting fungal structures in clear lactic acid and 30 measurements at ×1000 magnification were determined for each isolate using a Leica compound microscope (DM 2500) with interference contrast (DIC) optics. Descriptions, nomenclature and illustrations of taxonomic novelties are deposited in MycoBank (Crous et al. 2004a).

### DNA extraction, PCR amplification and sequencing

Genomic DNA was extracted from colonies grown on cellophane-covered PDA using a CTAB [cetyltrimethylammonium bromide] method (Doyle and Doyle 1990). DNA was estimated by electrophoresis in 1% agarose gel, and the quality was measured using the NanoDrop 2000 (Thermo Scientific, Waltham, MA, USA), following the user manual (Desjardins et al. 2009). PCR amplifications were performed in a DNA Engine Peltier Thermal Cycler (PTC-200; Bio-Rad Laboratories, Hercules, CA, USA). The primer set ITS1/ITS4 (White et al. 1990) was used to amplify the ITS region. The primer pair CAL228F/CAL737R (Carbone and Kohn 1999) was used to amplify the calmodulin gene (*cal*), and the primers CYLH4F (Crous et al. 2004b) and H3-1b (Glass and Donaldson 1995) were used to amplify part of the histone H3 (*his3*) gene. The primer pair EF1-728F/EF1-986R (Carbone and Kohn 1999) was used to amplify a partial fragment of the translation elongation factor 1-α gene (*tef1*). The primer set T1 (O’Donnell and Cigelnik 1997) and Bt2b (Glass and Donaldson 1995) was used to amplify the beta-tubulin gene (*tub2*); the additional combination of Bt2a/Bt2b (Glass and Donaldson 1995) was used in case of amplification failure of the T1/Bt2b primer pair. The PCR amplifications of the genomic DNA with the phylogenetic markers were done using the same primer pairs and conditions as in Yang et al. (2018). PCR amplification products were assayed via electrophoresis in 2% agarose gels. DNA sequencing was performed using an ABI PRISM 3730XL DNA Analyzer with a BigDye Terminater Kit v.3.1 (Invitrogen, USA) at the Shanghai Invitrogen Biological Technology Company Limited (Beijing, China).

### Phylogenetic analyses

The quality of the amplified nucleotide sequences was checked and combined using SeqMan v.7.1.0 and reference sequences were retrieved from the National Center for Biotechnology Information (NCBI), based on recent publications on the genus *Diaporthe* (Guarnaccia et al. 2018; Yang et al. 2018, 2020, 2021). Sequences were aligned using MAFFT v. 6 (Katoh and Toh 2010) and corrected manually using Bioedit 7.0.9.0 (Hall 1999). The best-fit nucleotide substitution models for each gene were selected using jModelTest v. 2.1.7 (Darriba et al. 2012) under the Akaike Information Criterion.

The phylogenetic analyses of the combined gene regions were performed using Maximum Likelihood (ML) and Bayesian Inference (BI) methods. ML was conducted using PhyML v. 3.0 (Guindon et al. 2010), with 1000 bootstrap replicates while BI was performed using a Markov Chain Monte Carlo (MCMC) algorithm in MrBayes v. 3.0 (Ronquist et al. 2003). Two MCMC chains, started from random trees for 1,000,000 generations and trees, were sampled every 100^th^ generation, resulting in a total of 10,000 trees. The first 25% of trees were discarded as burn-in of each analysis. Branches with significant Bayesian Posterior Probabilities (BPP) were estimated in the remaining 7500 trees. Phylogenetic trees were viewed with FigTree v.1.3.1 (Rambaut and Drummond 2010) and processed by Adobe Illustrator CS5. The nucleotide sequence data of the new taxa were deposited in GenBank (Table 1). The multilocus sequence alignments were deposited in TreeBASE (www.treebase.org) as accession S28703 and S22703.

**Table 1.** Isolates and GenBank accession numbers used in the phylogenetic analyses of *Diaporthe*.

Species	Isolate	Host	Location	GenBank accession numbers				
				ITS	*cal*	*his3*	*tef1*	*tub2*
*D.acericola*	MFLUCC 17-0956	*Acernegundo*	Italy	KY964224	KY964137	NA	KY964180	KY964074
*D.acerigena*	CFCC 52554	*Acertataricum*	China	MH121489	MH121413	MH121449	MH121531	NA
*D.alangii*	CFCC 52556	*Alangiumkurzii*	China	MH121491	MH121415	MH121451	MH121533	MH121573
*D.alnea*	CBS 146.46	*Alnus* sp.	Netherlands	KC343008	KC343250	KC343492	KC343734	KC343976
*D.amygdali*	CBS 126679	*Prunusdulcis*	Portugal	KC343022	KC343264	KC343506	AY343748	KC343990
*D.angelicae*	CBS 111592	*Heracleumsphondylium*	Austria	KC343027	KC343269	KC343511	KC343753	KC343995
*D.apiculatum*	CGMCC 3.17533	*Camelliasinensis*	China	KP267896	NA	NA	KP267970	KP293476
*D.arecae*	CBS 161.64	*Arecacatechu*	India	KC343032	KC343274	KC343516	KC343758	KC344000
*D.arengae*	CBS 114979	*Arengaenngleri*	Hong Kong	KC343034	KC343276	KC343518	KC343760	KC344002
*D.aseana*	MFLUCC 12-0299	Unknown dead leaf	Thailand	KT459414	KT459464	NA	KT459448	KT459432
*D.biguttulata*	CGMCC 3.17248	*Citruslimon*	China	KJ490582	NA	KJ490524	KJ490461	KJ490403
	CFCC 52584	*Juglansregia*	China	MH121519	MH121437	MH121477	MH121561	MH121598
***D.camelliae-oleiferae***	**HNZZ027**	***Camelliaoleifera***	**China**	**MZ509555**	**MZ504685**	**MZ504696**	**MZ504702**	**MZ504718**
	**HNZZ030**	***Camelliaoleifera***	**China**	**MZ509556**	**MZ504686**	**MZ504697**	**MZ504708**	**MZ504719**
	**HNZZ032**	***Camelliaoleifera***	**China**	**MZ509557**	**MZ504687**	**MZ504698**	**MZ504709**	**MZ504720**
*D.celeris*	CPC 28262	*Vitisvinifera*	Czech Republic	MG281017	MG281712	MG281363	MG281538	MG281190
*D.celastrina*	CBS 139.27	*Celastrus* sp.	USA	KC343047	KC343289	KC343531	KC343773	KC344015
*D.cercidis*	CFCC 52565	*Cercischinensis*	China	MH121500	MH121424	MH121460	MH121542	MH121582
*D.charlesworthii*	BRIP 54884m	*Rapistrumrugostrum*	Australia	KJ197288	NA	NA	KJ197250	KJ197268
*D.chrysalidocarpi*	SAUCC194.35	*Chrysalidocarpuslutescens*	China	MT822563	MT855646	MT855532	MT855876	MT855760
*D.cinnamomi*	CFCC 52569	*Cinnamomum* sp.	China	MH121504	NA	MH121464	MH121546	MH121586
*D.citriasiana*	CGMCC 3.15224	*Citrusunshiu*	China	JQ954645	KC357491	KJ490515	JQ954663	KC357459
*D.citrichinensis*	CGMCC 3.15225	*Citrus* sp.	China	JQ954648	KC357494	NA	JQ954666	NA
*D.collariana*	MFLU 17-2770	*Magnoliachampaca*	Thailand	MG806115	MG783042	NA	MG783040	MG783041
*D.conica*	CFCC 52571	*Alangiumchinense*	China	MH121506	MH121428	MH121466	MH121548	MH121588
*D.cucurbitae*	CBS 136.25	*Arctium* sp.	Unknown	KC343031	KC343273	KC343515	KC343757	KC343999
*D.cuppatea*	CBS 117499	*Aspalathuslinearis*	South Africa	KC343057	KC343299	KC343541	KC343783	KC344025
*D.discoidispora*	ZJUD89	*Citrusunshiu*	China	KJ490624	NA	KJ490566	KJ490503	KJ490445
*D.drenthii*	BRIP 66524	*Macadamia* sp.	South Africa	MN708229	NA	NA	MN696526	MN696537
*D.endophytica*	CBS 133811	*Schinusterebinthifolius*	Brazil	KC343065	KC343307	KC343549	KC343791	KC343065
*D.eres*	AR5193	*Ulmus* sp.	Germany	KJ210529	KJ434999	KJ420850	KJ210550	KJ420799
*D.fraxini-angustifoliae*	BRIP 54781	*Fraxinusangustifolia*	Australia	JX862528	NA	NA	JX862534	KF170920
*D.fraxinicola*	CFCC 52582	*Fraxinuschinensis*	China	MH121517	MH121435	NA	MH121559	NA
*D.fructicola*	MAFF 246408	*Passifloraedulis* × P.edulisf.flavicarpa	Japan	LC342734	LC342738	LC342737	LC342735	LC342736
*D.fusicola*	CGMCC 3.17087	*Lithocarpusglabra*	China	KF576281	KF576233	NA	KF576256	KF576305
*D.ganzhouensis*	CFCC 53087	Unknown	China	MK432665	MK442985	MK443010	MK578139	MK578065
*D.garethjonesii*	MFLUCC 12-0542a	Unknown dead leaf	Thailand	KT459423	KT459470	NA	KT459457	KT459441
*D.guangxiensis*	JZB320094	*Vitisvinifera*	China	MK335772	MK736727	NA	MK523566	MK500168
*D.helicis*	AR5211	*Hederahelix*	France	KJ210538	KJ435043	KJ420875	KJ210559	KJ420828
*D.heterostemmatis*	SAUCC194.85	*Heterostemmagrandiflorum*	China	MT822613	MT855692	MT855581	MT855925	MT855810
***D.hubeiensis***	JZB320123	*Vitisvinifera*	China	MK335809	MK500235	NA	MK523570	MK500148
	**HNZZ009**	***Camelliaoleifera***	**China**	**MZ509553**	**MZ504683**	**MZ504694**	**MZ504705**	**MZ504716**
	**HNZZ019**	***Camelliaoleifera***	**China**	**MZ509554**	**MZ504684**	**MZ504695**	**MZ504706**	**MZ504717**
***D.hunanensis***	**HNZZ023**	***Camelliaoleifera***	**China**	**MZ509550**	**MZ504680**	**MZ504691**	**MZ504702**	**MZ504713**
	**HNZZ025**	***Camelliaoleifera***	**China**	**MZ509551**	**MZ504681**	**MZ504692**	**MZ504703**	**MZ504714**
	**HNZZ033**	***Camelliaoleifera***	**China**	**MZ509552**	**MZ5046802**	**MZ504693**	**MZ504704**	**MZ504715**
*D.kadsurae*	CFCC 52586	*Kadsuralongipedunculata*	China	MH121521	MH121439	MH121479	MH121563	MH121600
*D.litchicola*	BRIP 54900	*Litchichinensis*	Australia	JX862533	NA	NA	JX862539	KF170925
*D.lonicerae*	MFLUCC 17-0963	*Lonicera* sp.	Italy	KY964190	KY964116	NA	KY964146	KY964073
*D.masirevicii*	BRIP 57892a	*Helianthusannuus*	Australia	KJ197277	NA	NA	KJ197239	KJ197257
*D.miriciae*	BRIP 54736j	*Helianthusannuus*	Australia	KJ197282	NA	NA	KJ197244	KJ197262
*D.momicola*	MFLUCC 16-0113	*Prunuspersica*	China	KU557563	KU557611	NA	KU557631	KU55758
*D.musigena*	CBS 129519	*Musa* sp.	Australia	KC343143	KC343385	KC343627	KC343869	KC344111
*D.neilliae*	CBS 144.27	*Spiraea* sp.	USA	KC343144	KC343386	KC343628	KC343870	KC344112
*D.nobilis*	CBS 113470	*Castaneasativa*	Korea	KC343146	KC343388	KC343630	KC343872	KC344114
*D.oraccinii*	CGMCC 3.17531	*Camelliasinensis*	China	KP267863	NA	KP293517	KP267937	KP293443
*D.ovoicicola*	CGMCC 3.17093	*Citrus* sp.	China	KF576265	KF576223	NA	KF576240	KF576289
*D.pandanicola*	MFLU 18-0006	*Pandanus* sp.	Thailand	MG646974	NA	NA	NA	MG646930
*D.pascoei*	BRIP 54847	*Perseaamericana*	Australia	JX862532	NA	NA	JX862538	KF170924
*D.passifloricola*	CBS 141329	*Passiflorafoetida*	Malaysia	KX228292	NA	KX228367	NA	KX228387
*D.penetriteum*	CGMCC 3.17532	*Camelliasinensis*	China	KP714505	NA	KP714493	KP714517	KP714529
*D.perseae*	CBS 151.73	*Perseagratissima*	Netherlands	KC343173	KC343415	KC343657	KC343899	KC344141
*D.pescicola*	MFLUCC 16-0105	*Prunuspersica*	China	KU557555	KU557603	NA	KU557623	KU557579
*D.pseudomangiferae*	CBS 101339	*Mangiferaindica*	Dominican Republic	KC343181	KC343423	KC343665	KC343907	KC344149
*D.pseudophoenicicola*	CBS 462.69	*Phoenixdactylifera*	Spain	KC343184	KC343426	KC343668	KC343910	KC344152
*D.pulla*	CBS 338.89	*Hederahelix*	Yugoslavia	KC343152	KC343394	KC343636	KC343878	KC344120
*D.racemosae*	CBS 143770	*Euclearacemosa*	South Africa	MG600223	MG600219	MG600221	MG600225	MG600227
*D.schimae*	CFCC 53103	*Schimasuperba*	China	MK432640	MK442962	MK442987	MK578116	MK578043
*D.schini*	CBS 133181	*Schinusterebinthifolius*	Brazil	KC343191	KC343433	KC343675	KC343917	KC344159
*D.schoeni*	MFLU 15-1279	*Schoenusnigricans*	Italy	KY964226	KY964139	NA	KY964182	KY964109
*D.searlei*	BRIP 66528	*Macadamia* sp.	South Africa	MN708231	NA	NA	NA	MN696540
*D.sennicola*	CFCC 51634	*Sennabicapsularis*	China	KY203722	KY228873	KY228879	KY228883	KY228889
*D.siamensis*	MFLUCC 10-573a	*Dasymaschalon* sp.	Thailand	JQ619879	NA	NA	JX275393	JX275429
***D.sojae***	FAU635	*Glycinemax*	USA	KJ590719	KJ612116	KJ659208	KJ590762	KJ610875
	**HNZZ008**	***Camelliaoleifera***	**China**	**MZ509547**	**MZ504677**	**MZ504688**	**MZ504699**	**MZ504710**
	**HNZZ010**	***Camelliaoleifera***	**China**	**MZ509548**	**MZ504678**	**MZ504689**	**MZ504700**	**MZ504711**
	**HNZZ022**	***Camelliaoleifera***	**China**	**MZ509549**	**MZ504679**	**MZ504690**	**MZ504701**	**MZ504712**
*D.spinosa*	PSCG	*Pyruspyrifolia*	China	MK626849	MK691129	MK726156	MK654811	MK691234
*D.sterilis*	CBS 136969	*Vacciniumcorymbosum*	Italy	KJ160579	KJ160548	MF418350	KJ160611	KJ160528
*D.subclavata*	ICMP20663	*Citrusunshiu*	China	KJ490587	NA	KJ490529	KJ490466	KJ490408
*D.subellipicola*	MFLU 17-1197	on dead wood	China	MG746632	NA	NA	MG746633	MG746634
*D.subordinaria*	CBS 464.90	*Plantagolanceolata*	New Zealand	KC343214	KC343456	KC343698	KC343940	KC344182
*D.taoicola*	MFLUCC 16-0117	*Prunuspersica*	China	KU557567	NA	NA	KU557635	KU557591
*D.tectonae*	MFLUCC 12-0777	*Tectonagrandis*	Thailand	KU712430	KU749345	NA	KU749359	KU743977
*D.tectonendophytica*	MFLUCC 13-0471	*Tectonagrandis*	Thailand	KU712439	KU749354	NA	KU749367	KU749354
*D.tectonigena*	MFLUCC 12-0767	*Tectonagrandis*	Thailand	KU712429	KU749358	NA	KU749371	KU743976
*D.terebinthifolii*	CBS 133180	*Schinusterebinthifolius*	Brazil	KC343216	KC343458	KC343700	KC343942	KC344184
*D.tibetensis*	CFCC 51999	*Juglandisregia*	China	MF279843	MF279888	MF279828	MF279858	MF279873
*D.tulliensis*	BRIP 62248a	*Theobromacacao*	Australia	KR936130	NA	NA	KR936133	KR936132
*D.ukurunduensis*	CFCC 52592	*Acerukurunduense*	China	MH121527	MH121445	MH121485	MH121569	NA
*D.unshiuensis*	CGMCC 3.17569	*Citrusunshiu*	China	KJ490587	NA	KJ490529	KJ490408	KJ490466
	CFCC 52594	*Caryaillinoensis*	China	MH121529	MH121447	MH121487	MH121571	MH121606
*D.viniferae*	JZB320071	*Vitisvinifera*	China	MK341551	MK500107	NA	MK500119	MK500112
*D.xishuangbanica*	CGMCC 3.18282	*Camelliasinensis*	China	KX986783	NA	KX999255	KX999175	KX999216
*D.yunnanensis*	CGMCC 3.18289	*Coffea* sp.	China	KX986796	KX999290	KX999267	KX999188	KX999228
*Diaporthellacorylina*	CBS 121124	*Corylus* sp.	China	KC343004	KC343246	KC343488	KC343730	KC343972

Note: NA, not applicable. Strains in this study are marked in bold.

## Results

### Phylogenetic analyses

The five-gene sequence dataset (ITS, *cal*, *his3*, *tef1* and *tub2*) was analysed to infer the interspecific relationships within *Diaporthe*. The dataset consisted of 96 sequences including the outgroup taxon, *Diaporthellacorylina* (CBS 121124). A total of 2520 characters including gaps (510 for ITS, 518 for *cal*, 533 for *his3*, 460 for *tef1* and 499 for *tub2*) were included in the phylogenetic analysis. The best nucleotide substitution model for ITS, *his3* and *tub2* was TrN+I+G, while HKY+I+G was selected for both *cal* and *tef1*. The topologies resulting from ML and BI analyses of the concatenated dataset were congruent (Fig. 1). According to the phylogenetic tree, two known species, *D.hubeiensis* and *D.sojae*, were part of *Diaporthe*.*Diaporthecamelliae-oleiferae* and *D.hunanensis* are new to science based on the distinct and well-supported molecular phylogenetic placement with their closest described relatives. Phylogenetically, *D.camelliae-oleiferae* clustered together with *D.pandanicola* and *D.viniferae*. *Diaporthehunanensis* clustered together with *D.chrysalidocarpi* and other species, including *D.drenthii*, *D.searlei* and *D.spinosa*.

**Figure 1.**
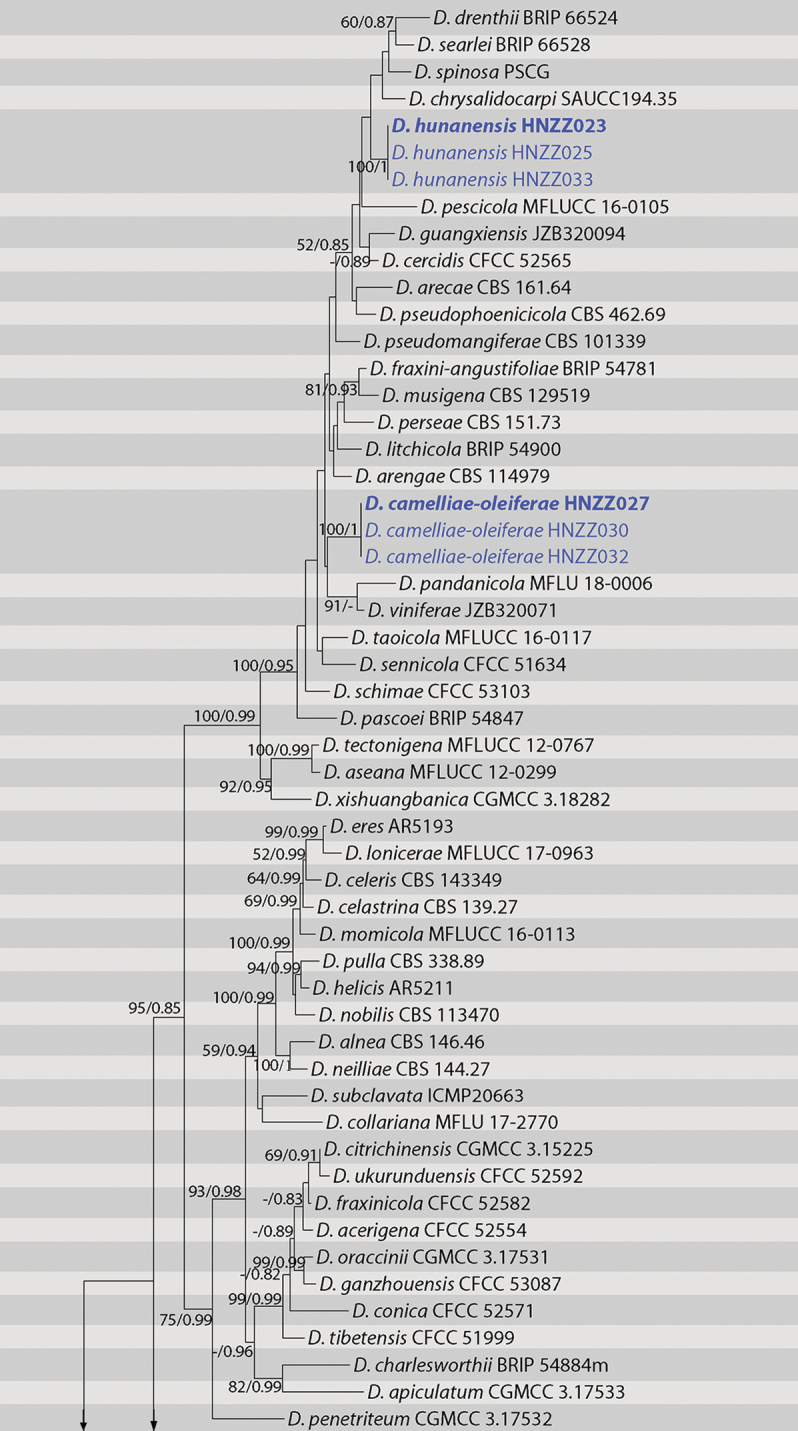
Phylogram of *Diaporthe* resulting from a maximum likelihood analysis based on combined ITS, *cal*, *his3*, *tef1* and *tub2*. Numbers above the branches indicate ML bootstraps (left, ML BS ≥ 50%) and Bayesian Posterior Probabilities (right, BPP ≥ 0.75). The tree is rooted with *Diaporthellacorylina*. Isolates in current study are in blue. “-” indicates ML BS < 50% or BI PP < 0.75.

**Figure 1.**
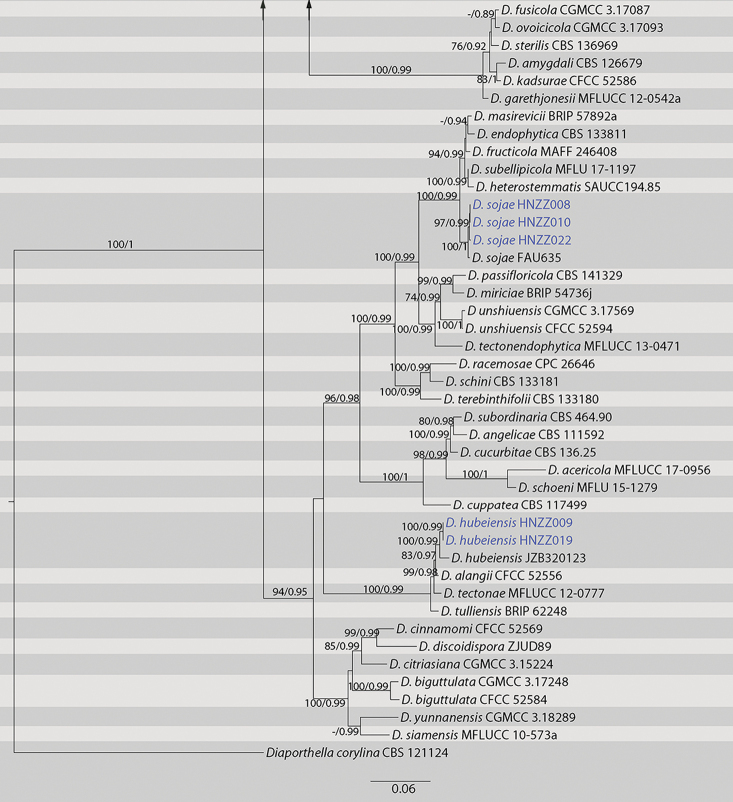
Continued

### Taxonomy

#### Diaporthe camelliae-oleiferae

Taxon classificationFungiDiaporthalesDiaporthaceae

Q. Yang sp. nov.

A6792E83-CF3B-58FC-92E0-E5A9400541DA

840451

Figure 2

##### Diagnosis.

Distinguished from the phylogenetically closely-related species, *D.pandanicola* and *D.viniferae* based on DNA sequence data.

##### Etymology.

Named after the host species, *Camelliaoleifera*.

##### Description.

Asexual morph: *pycnidia* on PDA 500–660 μm in diam., superficial, scattered on PDA, dark brown to black, globose, solitary, or clustered in groups of 3–5 pycnidia. Pale yellow conidial drops exuding from ostioles. *Conidiophores* reduced to conidiogenous cells. *Conidiogenous cells* (7.5–)10–14(–15.5) × 1.5–2.3 μm (n = 30), aseptate, cylindrical, straight, densely aggregated, terminal, slightly tapered toward the apex. *Alpha conidia* 5–6.5(–7.5) × 1.9–2.3 μm (n = 30), aseptate, hyaline, ellipsoidal to fusiform, biguttulate. *Beta conidia* (26.5–)28.5–31(–33) × 0.8–1.2 µm (n = 30), hyaline, aseptate, filiform, sinuous at one end, eguttulate.

**Figure 2.**
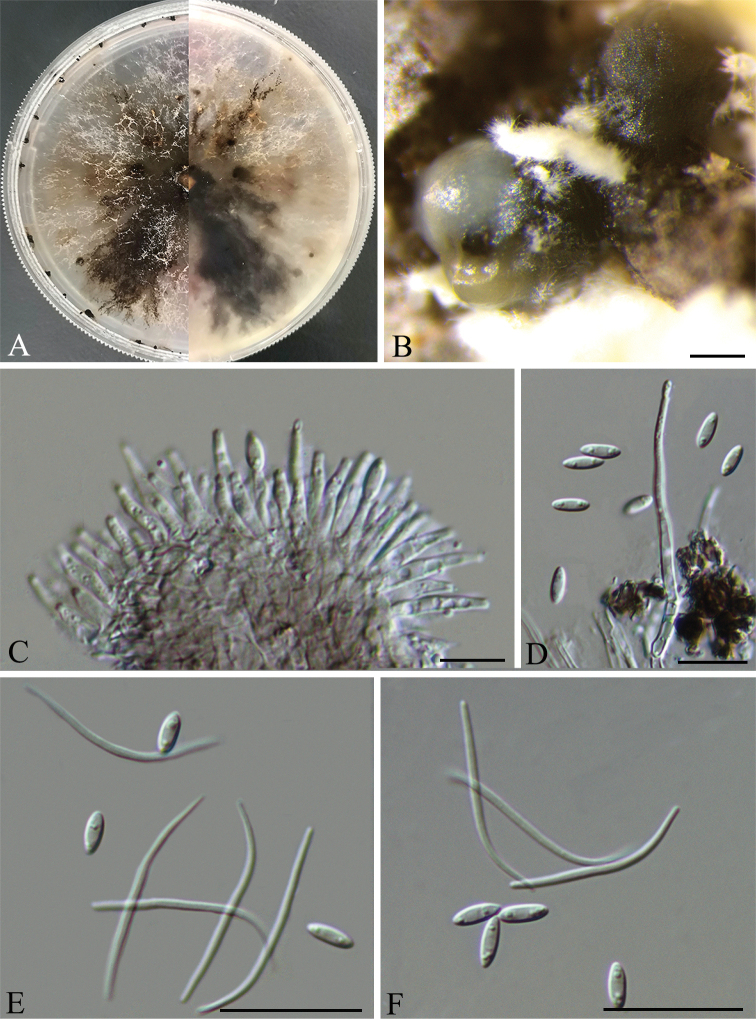
*Diaporthecamelliae-oleiferae* (HNZZ027) **A** Culture on PDA**B** conidiomata **C** conidiogenous cells **D–F** alpha and beta conidia. Scale bars: 200 μm (**B**); 10 μm (**C–D**); 20 μm (**E, F**).

##### Culture characters.

Culture incubated on PDA at 25 °C, originally flat with white fluffy aerial mycelium, becoming brown to black in the centre, with yellowish-cream conidial drops exuding from the ostioles after 20 days.

##### Specimens examined.

China. Hunan Province: Zhuzhou City, on leaves of *Camelliaoleifera*, 27°2'41"N, 113°19'17"E, 14 Aug. 2020, *Q. Yang* (holotype CSUFT027; ex-type living culture: HNZZ027; other living cultures: HNZZ030 and HNZZ032).

##### Notes.

Three isolates representing *D.camelliae-oleiferae* cluster in a well-supported clade (ML/BI=100/1) and appear most closely related to *D.pandanicola* on *Pandanus* sp. and *D.viniferae* on *Vitisvinifera*. *Diaporthecamelliae-oleiferae* can be distinguished from *D.pandanicola* based on ITS and *tub2* loci (24/462 in ITS and 11/401 in *tub2*); from *D.viniferae* based on ITS, *cal*, *tef1* and *tub2* loci (13/453 in ITS, 42/448 in *cal*, 7/339 in *tef1* and 26/402 in *tub2*). Morphologically, *D.camelliae-oleiferae* differs from *D.viniferae* in having shorter alpha conidia (5–6.5 μm vs. 5–8.3 μm) (Manawasinghe et al. 2019); from *D.pandanicola* in having narrower alpha conidia (1.9–2.3 μm vs. 2.5–3.2 μm) (Huang et al. 2021).

#### Diaporthe hubeiensis

Taxon classificationFungiDiaporthalesDiaporthaceae

Dissanayake, X.H. Li & K.D. Hyde

22A1496A-2AD0-5002-A1D7-7888283CD8FC

Figure 3

Manawasinghe, Dissanayake, Li, Liu, Wanasinghe, Xu, Zhao, Zhang, Zhou, Hyde, Brooks & Yan, Frontiers in Microbiology 10(no. 1936): 20 (2019)

##### Description.

Asexual morph: *pycnidia* on PDA in culture, 700–885 μm in diam., superficial, scattered, dark brown to black, globose or subglobose. *Conidiophores* reduced to conidiogenous cells. *Conidiogenous cells* (6.5–)7–10(–11.5) × 2–3.5 μm (n = 30), aseptate, cylindrical, phiailidic, straight or slightly curved. *Alpha conidia* 5.8–8(–8.5) × 2.5–3.2 μm (n = 30), aseptate, hyaline, ellipsoidal to cylindrical, biguttulate, blunt at both ends. *Beta conidia* not observed.

**Figure 3.**
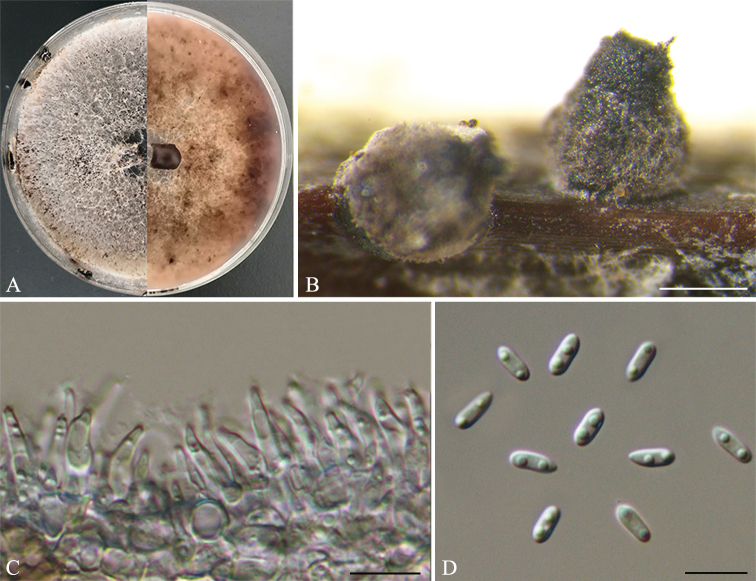
*Diaporthehubeiensis* (HNZZ019) **A** Culture on PDA**B** conidiomata **C** conidiogenous cells **D** alpha conidia. Scale bars: 500 μm (**B**); 10 μm (**C–D**).

##### Culture characters.

Culture incubated on PDA at 25 °C, originally flat with white felted aerial mycelium, becoming dark brown mycelium due to pigment formation, conidiomata irregularly distributed over agar surface after 20 days.

##### Specimens examined.

China. Hunan Province: Zhuzhou City, on leaves of *Camelliaoleifera*, 27°2'35"N, 113°19'20"E, 14 Aug. 2020, *Q. Yang* (CSUFT019; living cultures: HNZZ019 and HNZZ009).

##### Notes.

*Diaporthehubeiensis* was originally described as pathogen of grapevines in Hubei Province, China (Manawasinghe et al. 2019). In the present study, two isolates (HNZZ019 and HNZZ009) are closely related to *D.hubeiensis* in the combined phylogenetic tree (Fig. 1). The differences of nucleotides in the concatenated alignment (1/460 in ITS, 3/458 in *cal*, 1/320 in *his3* and 3/433 in *tub2*) are minor. Morphological comparison indicated that the isolates were similar to *D.hubeiensis* by the size of alpha conidia. We therefore identify the isolates as belonging to *D.hubeiensis*.

#### Diaporthe hunanensis

Taxon classificationFungiDiaporthalesDiaporthaceae

Q. Yang sp. nov.

00826C31-14C6-58BC-A164-2953E03882C1

840452

Figure 4

##### Diagnosis.

Distinguished from its phylogenetically closely-related species, *D.chrysalidocarpi*, *D.drenthii*, *D.searlei* and *D.spinosa* based on DNA sequence data.

##### Etymology.

In reference to the Hunan province, from where the fungus was first collected.

##### Description.

Asexual morph: *pycnidia* on PDA 180–300 μm in diam., superficial, scattered, black, globose, solitary in most. *Conidiophores* reduced to conidiogenous cells. *Conidiogenous cells* (8–)9–15(–16.5) × 1.7–2.1 μm (n = 30), aseptate, cylindrical, phiailidic, straight or slightly curved. *Alpha conidia* 6.5–7.5(–8.5) × 2.4–2.9 μm (n = 30), aseptate, hyaline, ellipsoidal, biguttulate, both ends obtuse. *Beta conidia* not observed.

**Figure 4.**
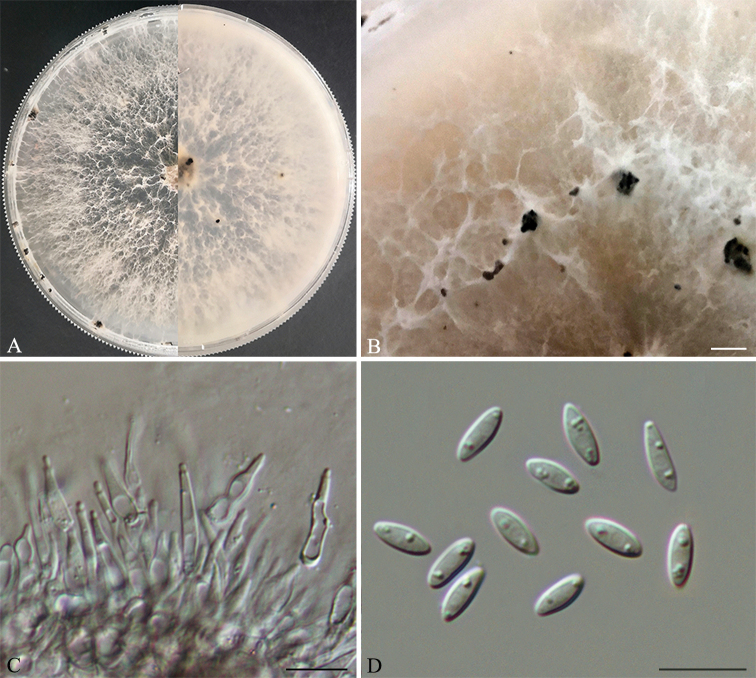
*Diaporthehunanensis* (HNZZ023) **A** Culture on PDA**B** conidiomata **C** conidiogenous cells **D** alpha conidia. Scale bars: 500 μm (**B**); 10 μm (**C–D**).

##### Culture characters.

Culture incubated on PDA at 25 °C, originally flat with white fluffy aerial mycelium, becoming pale brown with age, with visible solitary conidiomata at maturity after 18 days.

##### Specimens examined.

China. Hunan Province: Zhuzhou City, on leaves of *Camelliaoleifera*, 27°2'41"N, 113°19'17"E, 14 Aug. 2020, *Q. Yang* (holotype CSUFT 023; ex-type living culture: HNZZ023; living cultures: HNZZ025 and HNZZ033).

##### Notes.

Three isolates representing *D.hunanensis* cluster in a well-supported clade (ML/BI=100/1) and appear most closely related to *D.chrysalidocarpi* on *Chrysalidocarpuslutescens*, *D.drenthii* and *D.searlei* on *Macadamia* sp., and *D.spinosa* on *P.pyrifolia* cv. Cuiguan. *Diaporthehunanensis* can be distinguished from *D.chrysalidocarpi* based on ITS, *cal*, *his3* and *tub2* loci (7/457 in ITS, 28/448 in *cal*, 8/455 in *his3* and 5/401 in *tub2*); from *D.drenthii* based on ITS, *tef1* and *tub2* loci (9/457 in ITS, 13/328 in *tef1* and 23/401 in *tub2*); from *D.searlei* based on ITS and *tub2* loci (10/457 in ITS and 12/401 in *tub2*); from *D.spinosa* based on ITS, *cal*, *his3*, *tef1* and *tub2* loci (8/458 in ITS, 31/448 in *cal*, 5/455 in *his3*, 8/328 in *tef1* and 19/401 in *tub2*). Morphologically, *D.chrysalidocarpi* produces only beta conidia, while *D.hunanensis* produces alpha conidia (Huang et al. 2021); *D.hunanensis* differs from *D.drenthii* and *D.searlei* in wider alpha conidia (2.4–2.9 μm in *D.hunanensis* vs. 1.5–2.5 μm in *D.drenthii* vs. 1.5–2 μm in *D.searlei*) (Wrona et al. 2020); from *D.spinosa* in shorter alpha conidia (6.5–7.5 × 2.4–2.9 μm vs. 5.5–8 × 2–3.5 μm) (Guo et al. 2020). Therefore, we establish this fungus as a novel species.

#### Diaporthe sojae

Taxon classificationFungiDiaporthalesDiaporthaceae

Lehman, Ann. Mo. bot. Gdn 10: 128 (1923)

25538BA7-9C8C-57BA-94C3-CEBE0BBD8E21

Figure 5

##### Description.

Sexual morph: *perithecia* on pine needles in culture, black, globose, 250–500 μm in diam., densely clustered in groups, deeply immersed with elongated, tapering perithecial necks protruding through substrata, 525–800 μm. *Asci* unitunicate, 8-spored, sessile, elongate to clavate, (35–)37–42(–44.5) × (8–)10–11.5 μm (n = 30). *Ascospores* hyaline, two-celled, often 4-guttulate, with larger guttules at centre and smaller one at ends, elongated to elliptical, slightly or not constricted at septum, (9–) 9.5–11.5 × 2.7–4 μm (n = 30). Asexual morph not observed.

**Figure 5.**
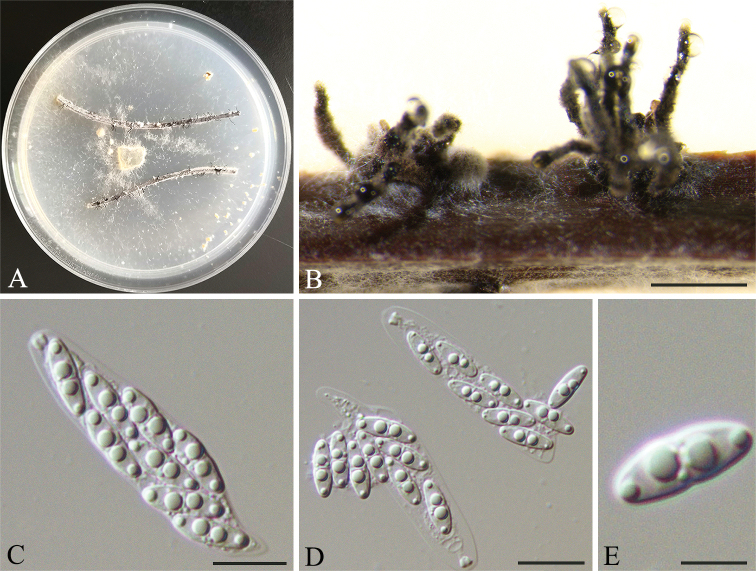
*Diaporthesojae* (HNZZ022) **A** Culture on PNA**B** ascomata **C–E** asci and ascospores. Scale bars: 500 μm (**B**); 10 μm (**C–E**).

##### Culture characters.

Culture incubated on PNA at 25 °C, originally white, fluffy aerial mycelium, reverse yellowish pigmentation developing in centre, later becoming dark brown, with yellowish-cream drops exuding from the perithecia after 15 days.

##### Specimens examined.

China. Hunan Province: Zhuzhou City, on leaves of *Camelliaoleifera*, 27°2'41"N, 113°19'17"E, 14 Aug. 2020, *Q. Yang* (USUFT 022; living cultures: HNZZ022, HNZZ008 and HNZZ010).

##### Notes.

*Diaporthesojae* was ﬁrst reported on pods and stems of soybean, and subsequently reported on a wide range of hosts (Dissanayake et al. 2015; Udayanga et al. 2015; Guo et al. 2020). It was also reported on some fruit trees in China, such as *Vitis* spp. (Dissanayake et al. 2015) and *Citrus* spp. (Huang et al. 2015). In the present, three isolates (HNZZ008, HNZZ010 and HNZZ022) are closely related to *D.sojae* in the combined phylogenetic tree (Fig. 1). The differences of nucleotides in the concatenated alignment (1/460 in ITS, 3/458 in *cal*, 1/320 in *his3* and 3/433 in *tub2*) are minor. Compared with the description of the ex-type isolate FAU635, the isolate has wider asci (10–11.5 μm vs. 7–9 μm) (Udayanga et al. 2015). We therefore identify the isolates as belonging to *D.sojae*.

## Discussion

In this study, an important oil-tea tree species, *Camelliaoleifera* was investigated and *Camellia* leaf disease was found as a common disease in plantations in Hunan Province. Identification of our collections was conducted, based on isolates from symptomatic leaves of *C.oleifera* using five combined loci (ITS, *cal*, *his3*, *tef1* and *tub2*), as well as morphological characters. It includes *D.hubeiensis*, *D.sojae*, as well as two new species named *D.camelliae-oleiferae* and *D.hunanensis*.

The expanding cultivation of *C.oleifera* over the last several decades has attracted increasing attention from plant pathologists to infectious diseases on this crop. Therein, diseases caused by *Diaporthe* species have becoming the emerging Camellia leaf diseases in southern China (Gao et al. 2016; Guarnaccia et al. 2018; Yang et al. 2018; Zhou and Hou 2019). Understanding the diversity of *Diaporthe* species and the genetic variation within pathogen populations could help in developing sustainable disease management strategies.

According to the USDA Fungal–host interaction database, there are two records of *Diaporthe* species associated with *C.oleifera* (https://nt.ars-grin.gov/fungaldatabases/fungushost/fungushost.cfm) (accessed 9 September 2021). These records are related to the following two *Diaporthe* species: *D.eres* and *D.huangshanensis* (Zhou and Hou 2019). *Diaportheeres*, the type species of the genus, was described by Nitschke (1870) on *Ulmus* sp. collected in Germany, which has a widespread distribution and a broad host range as pathogens, endophytes or saprobes (Udayanga et al. 2014b). *Diaportheeres* differs from *D.camelliae-oleiferae* and *D.hunanensis* in having wider alpha conidia (3–4 μm in *D.eres* vs. 1.9–2.3 μm in *D.camelliae-oleiferae* vs. 2.4–2.9 μm in *D.hunanensis*) (Gomes et al. 2003); *D.huangshanensis* differs from *D.camelliae-oleiferae* in having larger alpha conidia (5.7–8.4 × 2.7–4.5 μm vs. 5–6.5 × 1.9–2.3 μm); from *D.hunanensis* in having wider alpha conidia (2.7–4.5 μm vs. 2.4–2.9 μm) and longer conidiophores (12.1–23.5 μm vs. 9–15 μm) (Zhou and Hou 2019).

As the species concept of *Diaporthe* has been improved a lot by using molecular data (Huang et al. 2015; Gao et al. 2016, 2017; Guarnaccia and Crous 2017; Guarnaccia et al. 2018; Yang et al. 2018, 2020, 2021; Manawasinghe et al. 2019; Guo et al. 2020), many new species have been discovered and reported in recent years. In this study, the *Diaporthe* isolates from *C.oleifera* were identified based on sequence analysis and morphological characteristics. Future studies should focus on pathogenicity, epidemiology and fungicide sensitivity of the important plant fungal pathogen to develop effective management of *C.oleifera* disease and on the pathogenic molecular mechanism.

## Supplementary Material

XML Treatment for Diaporthe camelliae-oleiferae

XML Treatment for Diaporthe hubeiensis

XML Treatment for Diaporthe hunanensis

XML Treatment for Diaporthe sojae
